# Impact of Microbial Metabolites on Microbiota–Gut–Brain Axis in Inflammatory Bowel Disease

**DOI:** 10.3390/ijms22041623

**Published:** 2021-02-05

**Authors:** Davide Banfi, Elisabetta Moro, Annalisa Bosi, Michela Bistoletti, Silvia Cerantola, Francesca Crema, Fabrizio Maggi, Maria Cecilia Giron, Cristina Giaroni, Andreina Baj

**Affiliations:** 1 Department of Medicine and Surgery, University of Insubria, via H Dunant 5, 21100 Varese, Italy; davide.banfi@uninsubria.it (D.B.); a.bosi@uninsubria.it (A.B.); m.bistoletti1@uninsubria.it (M.B.); fabrizio.maggi@uninsubria.it (F.M.); andreina.baj@uninsubria.it (A.B.); 2 Department of Internal Medicine and Therapeutics, Section of Pharmacology, University of Pavia, via Ferrata 9, 27100 Pavia, Italy; elisabetta.moro@unipv.it (E.M.); francesca.crema@unipv.it (F.C.); 3 Department of Pharmaceutical and Pharmacological Sciences, University of Padova, Largo Meneghetti 2, 35131 Padova, Italy; silvia.cerantola@unipd.it (S.C.); cecilia.giron@unipd.it (M.C.G.); 4 Centre of Neuroscience, University of Insubria, 21100 Varese, Italy

**Keywords:** IBD, microbiota–gut–brain axis, dysbiosis, microbiota targeting therapies, antibiotics, probiotics, prebiotics, fecal transplant therapy

## Abstract

The complex bidirectional communication system existing between the gastrointestinal tract and the brain initially termed the “gut–brain axis” and renamed the “microbiota–gut–brain axis”, considering the pivotal role of gut microbiota in sustaining local and systemic homeostasis, has a fundamental role in the pathogenesis of Inflammatory Bowel Disease (IBD). The integration of signals deriving from the host neuronal, immune, and endocrine systems with signals deriving from the microbiota may influence the development of the local inflammatory injury and impacts also more distal brain regions, underlying the psychophysiological vulnerability of IBD patients. Mood disorders and increased response to stress are frequently associated with IBD and may affect the disease recurrence and severity, thus requiring an appropriate therapeutic approach in addition to conventional anti-inflammatory treatments. This review highlights the more recent evidence suggesting that alterations of the microbiota–gut–brain bidirectional communication axis may concur to IBD pathogenesis and sustain the development of both local and CNS symptoms. The participation of the main microbial-derived metabolites, also defined as “postbiotics”, such as bile acids, short-chain fatty acids, and tryptophan metabolites in the development of IBD-associated gut and brain dysfunction will be discussed. The last section covers a critical evaluation of the main clinical evidence pointing to the microbiome-based therapeutic approaches for the treatment of IBD-related gastrointestinal and neuropsychiatric symptoms.

## 1. Introduction

Inflammatory bowel diseases (IBD) comprise two diagnostically distinct, but pathologically similar disorders: Crohn’s disease (CD) and ulcerative colitis (UC), both manifesting as chronic and relapsing inflammatory disorders [1]. In CD, inflammation injury develops along the whole gastrointestinal tract, while in UC the inflammatory response is limited to the rectum and colon [2]. Since the middle of the last century, IBD has become a public health burden with increasing incidence in the western world, where its prevalence has plateaued at the beginning of the twenty-first century, while it is gradually becoming more common in developing countries [1,3]. Both CD and UC are characterized by acute intestinal inflammatory flares of extremely variable duration and frequency, associated with spontaneously occurring or treatment-induced intervals of remission. The specific etiopathogenetic mechanism at the basis of IBD remains to be fully elucidated, although there is consensus on the involvement of an exaggerated immune response induced by different triggering stimuli deriving from the gut microbiota, pathogenetic microorganisms, or from the environment in a genetically susceptible host [4,5].

Main symptoms observed in IBD patients originate from the altered sensory, motor, and secretory intestinal functions, resulting in abdominal pain, diarrhea, gastrointestinal bleeding, and malnutrition [6]. Two principal phases are recognized in the development of the intestinal damage: a transitory acute phase characterized by prominent epithelial injury, altered secretion, and enhanced visceral sensitivity, which is followed by long-term changes involving the neuromuscular layer, with persistent alteration of the motor function [7,8]. This latter response involves structural and functional changes within the enteric nervous system (ENS) as a result of the interplay taking place between enteric neurons and different cellular populations of the gut microenvironment, infiltrating immunocytes and the saprophytic microflora [7,8,9]. The enteric microbiota is considered a fundamental player in the development of IBD, influencing both the acute phase and the more latent inflammation-induced neuronal dysfunction. Indeed, the correlation between gut dysbiosis, i.e., changes in intestinal microbial composition, and IBD etiopathogenesis has been consistently shown both in preclinical and clinical studies [4,10]. In addition to intestinal symptoms, IBD patients present with extra-intestinal stress-related disorders, such as major depression and generalized anxiety, which influence the outcome of disease treatment reducing the chance of remission [11,12,13,14,15]. IBD patients suffering from psychiatric disorders are at higher risk of requiring surgery and of incurring functional gut disorders [16]. The Manitoba IBD cohort study reports that persons with IBD display significantly higher lifetime prevalence of both depression and some anxiety disorders with respect to a matched community sample without the disease [17]. Pediatric IBD patients, presenting with neuropsychiatric symptoms often associated with mild behavioral and cognitive disturbances, particularly verbal memory loss, typically develop a more severe disease state and poor quality of life as compared to patients without these comorbidities [18,19,20]. The exact mechanism/s underlying the association between the severity of IBD and psychiatric disorders have not been unveiled yet, although there are hints suggesting that they may relate to alterations of the saprophytic microflora composition and function, resulting in changes along the so-called “microbiota–gut–brain” [11,21,22]. The majority of studies carried out to delineate the role of gut–brain interactions in the context of gastrointestinal diseases have focused on functional disorders, such as irritable bowel syndrome (IBS) [23]. However, the correlation between IBD and dysregulated gut–brain communication is now acknowledged [24]. This bidirectional communication axis allows signals originating within the central nervous system (CNS) to control gut motor, sensory, secretory, and immune functions. Conversely, messages from the gastrointestinal tract may be conveyed to brain regions, where these signals may influence high functions such as behavior, cognition and emotion regulation, both in normal and disease states [25,26,27]. The gut microbiota is a fundamental player in gut–brain communication by releasing a plethora of metabolites, which are involved in the control of local metabolic, neuronal, and immune functions and extend their actions to more distal regions within the CNS, being critical for brain development and homeostasis [25,26,27]. In this context, changes within the symbiotic interplay between the host and the saprophytic microflora during IBD may bear important consequences for the host health, underlying not only development of gastrointestinal symptoms but also favoring the patient psychophysiological vulnerability [22,24,28]. This review encompasses a novel and comprehensive overview of the more recent evidences suggesting that alterations in the microbiota–gut–brain bidirectional communication pathways may concur to IBD pathogenesis, sustaining the development of both local and CNS symptoms. In this context, we will consider the relevance of the main microbial-derived metabolites, also defined as “postbiotics”, such as bile acids, short-chain fatty acids, and tryptophan metabolites in the development of IBD-associated gut and brain dysfunctions. The last section covers a critical evaluation of the main available clinical evidence pointing to the manipulation of the saprophytic gut microflora as a possible adjuvant approach for the treatment of IBD-related gastrointestinal and neuropsychiatric symptoms.

## 2. The Gut Microbiota and IBD

The gut microbiota is composed of a rich and dynamic population of saprophytic microorganisms mainly composed of Bacteria, represented by 3.8 × 1013 cells, 2–4 million genes, more than 1000 species, and 7000 strains, but comprising also Viruses, Archaea, Fungi, and Protozoa [29,30]. This vast microbial community has adapted to the host gut microenvironment along congruent phylogenetic pathways leading to a composite mammalian host-gut microbiota entity [31]. The existence of a symbiotic relationship between the gut microbiota and the host is progressively being elucidated by resorting to sophisticated metagenomic, metabolomic, and proteomic approaches as well as to the availability of numerous preclinical animal models including germ-free (GF) and dysbiotic animal models [27]. The gut microbiota plays a fundamental role in the maintenance of the host health by modulating immune responses, inducing defense mechanisms against pathogens, promoting the fermentation of indigestible dietary fibers, vitamin synthesis, and drug metabolism [25,26,27]. Although the microbiota displays some resilience to unstable environmental conditions occurring, for example, as a result of diet modifications or short-term antibiotic treatment [32,33], the combination of several factors such as consumption of high-fat food, drugs, age, and genetics may alter the microflora composition and function, hampering the positive relationship with the host [34,35]. It is now ascertained that an unbalanced composition of the gut microflora, promoted by some of these factors, may contribute to the etiopathogenesis of IBD [4,5]. While an increasing number of studies suggest that all the gut microbial components, including Bacteria, Virus, Fungi, Archae, and Helmints may play a causative role in IBD development and chronicization, for the sake of simplicity, this dissertation will encompass only the pathogenetic role of the bacterial microbiota [4,36]. The healthy gut bacterial community is mainly represented by the phyla *Firmicutes*, *Bacteroidetes*, *Actinobacteria*, and Verrucomicrobia [37] although significant inter-individual microbial diversity has been demonstrated within these major phenotypes [38]. Metagenomic studies point to a significant alteration of this microbial pattern in UC and CD patients, characterized by high load and less diversity and intestinal dysbiosis is reported to occur in 70% of naïve treatment IBD patients, and in 80% of patients with quiescent IBD, as compared to the 16% of healthy subjects [39,40]. Indeed, in IBD patients, microbial instability has been demonstrated in terms of both reduced biodiversity and richness in the number of species within a community, which may be circumscribed to the inflamed regions in CD patients and relates to the disease activity index [41,42,43]. 

The pattern of dysbiosis in IBD patients is mainly characterized by a reduction in the abundance of bacterial species within the Phyla of *Firmicutes* and *Bacteroidetes* and a relative increase of bacterial species belonging to the Enterobacteriaceae family, within the Phylum of Proteobacteria [36] (Table 1). Specific pathobionts, e.g., commensal microorganisms that in definite environmental or genetic conditions can cause the disease, however, have not been identified yet [44]. Interestingly, a recent taxonomic study resorting to public gut microbiome data of fecal samples and biopsies obtained from IBD and healthy individuals to reconstruct a bacterial family co-abundance network shows that bacterial families which have an increased abundance level in IBD conditions are not well connected to other bacterial groups in the global family co-abundance network, suggesting that these bacteria do not co-exist with healthy gut microbial commensals [45].

Many studies address the attention to the high levels of particularly adherent-invasive strains of *Escherichia coli* (*E. Coli* AIEC) within the *Enterobacteriaceae* family, which were found in ileal biopsies of patients with active CD [46,47,48] as well as in UC patients [49]. *Fusobacterium* represents another adherent-invasive bacteria genus, which has been found in high amounts in mucosal specimens of UC patients and is proposed to influence IBD development [36]. Ohkusa et al. demonstrated that rectal enema administration of supernatants from *Fusobacterium varium* isolated from the inflamed mucosa of UC patients could induce colonic inflammation in mice [50]. Conversely, several other bacterial species are considered to play a beneficial role against IBD, such as *Lactobacillus,* and *Faecalibacterium* within *Firmicutes*, and *Bifidobacterium* within *Actinobacteria* [36]. In ileal biopsies from CD patients, an unbalanced bacterial composition, with significantly reduced *Faecalibacterium prausnitzii (F. prausnitzii)* levels and increased *E.Coli* levels was related to the disease phenotype, since it could not be evidenced in colonic CD biopsies [51,52]. *F. prausnitzii,* an SCFA-producing species belonging to *Clostridium* cluster IV, is considered an anti-inflammatory species, protecting the host mucosa from inflammation injury by favoring the production of anti-inflammatory cytokines, such as IL-10 [53]. In CD patients, reduced amounts of *F. prausnitzii* were associated with an increased risk of recurrence after surgery, while in UC patients, clinical remission could be assured by the restoration of this species [52,54]. Reduced levels of SCFA-producing bacteria strains in *Clostridium* clusters IV, XIVa in fecal samples from IBD patients represent another consistent clinical data [55]. Accordingly, in a recent study, the increased risk of CD recurrence after bowel resection was associated with enriched diversity in the *Enterobacteriaceae* family, whilst maintenance of remission was associated with increased diversity in the *Lachnospiraceae* family, which reside within the *Clostridium* clusters, XIVa [56].

The relationship between IBD and dysbiosis is, however, complex and it has not been clear-cut clarified yet if gut microbiota changes represent a cause or a consequence of the disease, as emerged in a large systematic review of case-control studies comparing gut microbiota in patients with IBD (CD and/or UC) and normal healthy controls [57]. According to some studies, dysbiosis may not represent a triggering factor for IBD but would probably evolve in a successive phase of the disease, possibly as a consequence of inflammation-induced changes in the enteric microenvironment and contributing to disease chronicization. Several evidences suggest that metabolic changes reflecting the environmental stress response during inflammation may favor the growth of a complex community of dysbiotic species [4]. Inflammation promotes mucosal oxidative stress, which may shape the indigenous microbial community favoring the growth of facultative anaerobes aerotolerant, such as *Ruminococcus gnavus*, which is considered an important member of the altered microbial gut community in IBD [58]. Small electron acceptor molecules produced during inflammation may underlay the bloom of facultative anaerobic microorganisms, such as Enterobacteriaceae, associated with IBD [59]. Conversely, other observations point to the hypothesis that dysbiosis may precede the disease, as suggested by quantitative and qualitative microbial changes observed in luminal and mucosal biopsies from new-onset untreated large pediatric CD cohorts [41]. Similarly, in a smaller prospective study of CD pediatric patients, dysbiosis reflected the presence and severity of inflammation; and was independently influenced by other factors such as diet and antibiotic exposure [60]. The first three years of life are critical for the establishment of a healthy and stable microbiota, and perturbations of the microbial community may have a significant impact on the developing individual with long-lasting effects on the host immune, neuronal and metabolic functions [27,61]. Caesarean delivery, formula nutrition, frequent antibiotic treatments, and living in highly sanitized environments are considered as risk factors for pediatric IBD development by promoting alterations in the dynamic microbiota–host relationship [4,27,61]. The consequences of early exposure to some of these risk factors may extend from the gut to the brain via microbiota–gut–brain signaling leading to neurodevelopmental disorders [62]. These considerations may be extended to adult age, when if unperturbed, the microbiota is relatively stable, but may be altered by many factors such as diet, genetics, geography, health or disease status, drugs, hygienic conditions, and lifestyle, possibly favoring the development of gut and brain disorders [63]. Interestingly, a recent, wide prospective case-control study carried out in IBD patients from adolescence to >65 years of age has evidenced a significant correlation between higher cumulative exposure to systemic antibiotic therapy and the risk of both UC and CD new-onset, further emphasizing the close relationship between microbiota perturbations and the development of IBD [64].

## 3. The Microbiota–Gut–Brain Axis and IBD

**Figure 1:**
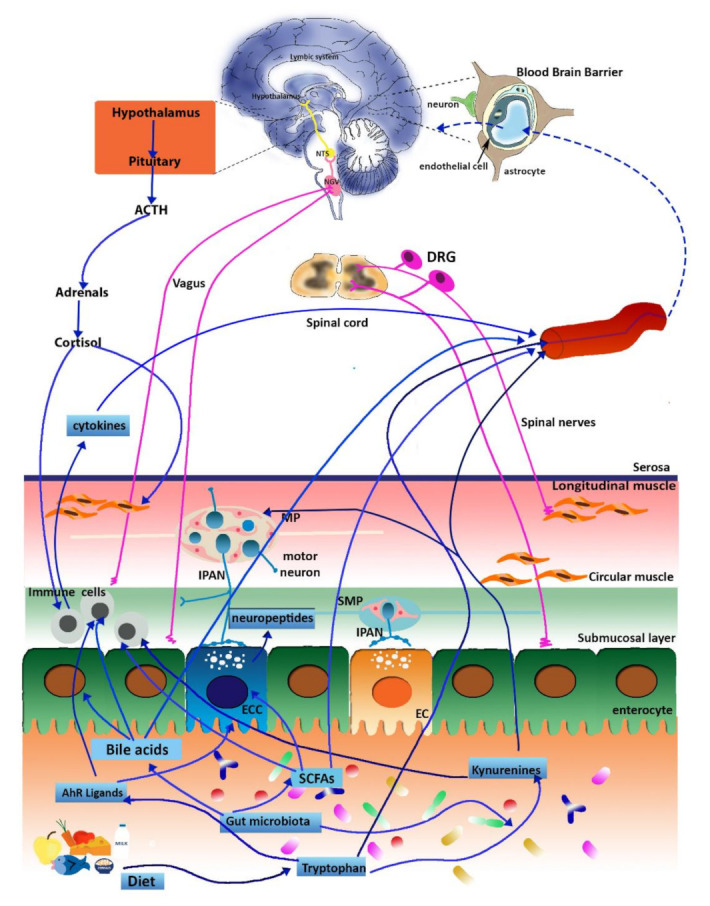
Schematic representation of the microbiota–gut–brain axis. Signals from the gut saprophytic microflora reach the central nervous system (CNS) and the enteric nervous system (ENS) via different pathways, including endocrine, immune, metabolic, and neuronal pathways as described in the text. In normal conditions, the blood–brain barrier allows the access of tryptophan, kynurenines, SCFAs, and bile acids. Abbreviations: NTS, the nucleus of the solitary tract; NVG, nodose vagal ganglion; DRG, dorsal root ganglion; MP, myenteric plexus; IPAN, intrinsic primary afferent neurons; SMP, submucosal plexus; ECC, enteroendocrine cell; EC, enterochromaffin cells; SCFAs, short-chain fatty acids (adapted from Baj et al., 2019 [26]).

A complex neural and hormonal reflex network participates in the formation of the gut–brain axis, allowing a bidirectional communication system between the gut and the brain, which are in continuous cross-talking both in health and disease states [25,26] (Figure 1). The anatomic-physiologic conduit of the gut–brain axis is represented by neural pathways of the autonomic nervous system (ANS), the ENS, hormonal (i.e., the hypothalamic-pituitary-adrenal, HPA, axis), and humoral systems [25]. Afferent neurons, within the parasympathetic (vagal) have their cell bodies in the nodose vagal ganglion (NVG) and transmit sensory information to the nucleus of the solitary tract (NTS) in the brain stem, and then, to higher centers, such as the paraventricular nucleus of the hypothalamus, or, more locally, inducing long vago-vagal reflexes [24,89]. Efferent vagal pathways drive signals to the periphery via the dorsal motor nucleus of the vagus (DMV) and the nucleus ambiguous (NA) in the brain stem, participating in the control of motor and secretory gut functions and of food intake [24,89]. Afferent spinal neurons, whose cell bodies are contained in dorsal root ganglia (DRG), transmit sensory inputs to dorsal horn neurons of the thoracic and upper lumbar spinal cord, which then project to the CNS via spinothalamic pathways and represent the main pain signaling pathways along the gut–brain axis [24,90]. In the CNS, both spinal and vagal afferent inputs synapse with higher brain regions, such as the emotional motor system, consisting of the limbic system and some paralimbic structures (including the medial prefrontal cortex, amygdala, and hypothalamus), involved in the control of emotional responses [24,90,91]. The ENS, a complex neuronal network innervating the gastrointestinal tract, represents a fundamental peripheral neuronal component, which receives sensory inputs from the ANS and transmits information to it [26,91]. In recent years, a vast number of preclinical and clinical studies have highlighted the fundamental contribution of the enteric microbiota in the formation of the “microbiota–gut–brain axis” in health and disease states [25,27]. In a seminal metabolomic study, an altered metabolic profile was demonstrated in GF mice as compared to their conventionalized ex-GF, with 10 bacterial metabolites involved in CNS signaling, supporting the hypothesis that gut microbes may influence high brain functions [92]. Although much of the mechanisms remains to be elucidated owing to the vast and diverse gut microbial composition, it is now clear that several microbially-derived molecules may influence different gut functions, including metabolic, nutritional, and immune responses, but also brain activity, giving rise to a microbiota-mediated bottom-up control of the CNS [27]. In this context an increasing amount of data is available to suggest that changes in the reciprocal interplay between the microbiota and the host gut–brain axis may participate in the manifestation of IBD symptoms [11,93]. In this context, this review aims to give an unprecedented comprehensive mechanistic description of the importance of different gut–brain axis communication pathways and their modulation by bacterial metabolites in the development of IBD-associated gastrointestinal and brain symptoms.

### 3.1. The Gut Immune System

The gut mucosal immune system participates in the maintenance of intestinal homeostasis by protecting the intestinal mucosa from exposure to pathogenic microbes by means of highly effective innate and adaptive immune mechanisms [94]. The gut mucosal layer represents a vast interface between the host and the outer environment and, as such, has a vital homeostatic role sustaining the host’s health status. However, under pathological conditions, such as during infection and inflammation, the barrier integrity may be compromised, becoming leaky and allowing the translocation of pathological bacteria across the mucosal lining. Barrier deterioration may allow microorganisms and/or their by-products to come in contact with cells of the enteric microenvironment, including immune cells [25]. The consequent activation of an immune response is favored by the commensal microbiota and is characterized by secretion of proinflammatory mediators, such as TNFα, IL-22, and IL-17, and activation of the inflammasome for host defense [95]. An overt dysbiotic microflora associated with IBD leads to host immune system dysfunction and the consequent development of chronic inflammation. The hypothesis that a particular microbial antigenic determinant may concur to the development of colitogenic changes in the intestinal mucosa has been demonstrated in a vast number of preclinical studies on IBD [96,97,98,99]. Several reports from clinical investigations and preclinical studies carried out on gnotobiotic animals suggest that specific microbial strains may drive Th1 and Th17-pathogenic immune responses [4]. The pathological expansion of Th17 with the consequent release of IL-17, is sustained by IL-23 secretion from dendritic cells. IL-23 is considered a key factor at the basis of IBD pathogenesis, and anti-IL23 agents are now developed with promising results for IBD therapy [100,101]. In IBD patients, the mechanisms involved in the process of oral tolerance are also defective, with low mucosal levels of the anti-inflammatory cytokine, IL-10, leading to the maturation of dendritic cells and the stimulation of Th1 pro-inflammatory responses [102,103]. IL-10 gene and TGF-β and IL-35 are proposed as candidate genes at the basis of IBD pathophysiology [104]. IL-10 polymorphisms have been associated with IBD in genome-wide association studies (GWAS), and mutations in IL-10 and IL-10 receptor genes have been reported in immunodeficient children with severe pediatric-onset IBD [105]. IL-10 is produced by Treg lymphocytes and mice overexpressing IL-23 or carrying a deletion for IL-10 develop a spontaneous inflammation, which strictly depends upon the composition of microbiota [105,106]. Overall, the microbiota, by regulating the relative abundance of Th17 and Treg, has a crucial role in driving the host organism towards either an inflammatory or a tolerogenic response [4]. In mice, activation of Foxp^3+^ Treg lymphocytes and production of TGF-β was favored by oral administration of a defined mix of *Clostridium* strains, resulting in the development of resistance to colitis [107]. The complex interplay between the host and the microbiota in the development of gut inflammatory responses involves also pathogen-associated molecular pattern receptors (PAMP) receptors, such as Toll-like receptors (TLR) and Nucleotide-binding oligomerization domain-like receptors (NLRs) of the innate immunity [4]. In different mutant murine models, experimentally-induced colitis was correlated with TLR signaling resulting in improved epithelial cell survival, inhibition of apoptosis, and recruitment of stromal and myeloid cells [108,109]. Transgenic mice carrying a mutation for intracellular molecular pathways downstream to TLRs as well as for other genes involved in the inflammatory response (NLRP3, NLRP6, Caspase1), display defective epithelial repair mechanisms and a higher predisposition to develop cancer. In these animals, the growth and translocation of pathogens are favored, suggesting that the microbiota, by modulating the innate immunity function, may sustain intestinal homeostasis, either by indirectly protecting the epithelial barrier or by reducing pathogen translocation [110,111,112]. Interestingly, in the context of an interkingdom cross-talk, butyrate treatment upregulated the expression of TLR4 in human colon cancer cell lines in vitro, *thus favoring the* priming *of innate* immune response and providing a mechanism against tumor growth [113]. The translational importance of these observations is emphasized by the observation that TLR polymorphisms/mutations have been identified and directly linked to IBD etiopathogenesis influencing the microbiota stability and immune response in IBD patients [114]. This opens the exciting scenario for the possible discovery of modulators of TLRs and of TLRs downward signaling pathways for potential therapeutic applications [114].

Another important consideration in the context of chronic inflammatory gut diseases is that in the gut, mucosal immunocytes are close to neuronal cells of the ENS and may regulate one another’s functions by releasing a complex set of cytokines, neurotransmitters, and hormones. Receptors for neuropeptides released by enteric nerves are present on lymphocytes, whose stimulation by SP or VIP can induce their differentiation and alter immunoglobulin production; on the reverse, it is now well ascertained that neuronal activity may be regulated by TLRs [115,116]. During chronic inflammation, this neuroimmune interplay is amplified leading to neuroimmune plasticity with neuronal and immune system structural and functional remodeling [117]. Such neuroimmune crosstalk, however, is not confined to the gut environment and extends to the ANS and CNS along the microbiota–gut–brain communication axis, as discussed in the next paragraphs. In this perspective, restoring impaired immune responses in the host by correcting dysbiosis and defective microbial metabolism may represent an innovative and safe preventive and/or therapeutic strategy to ameliorate gut and brain disorders associated with overt colitis [94].

### 3.2. The ENS

The ENS extends from the esophagus to the anal sphincter forming a complex network of ganglia and interconnecting fiber strands, which may respond directly, or indirectly, to several gut microbiota-derived metabolites [26,118]. The ENS innervates epithelial cells, blood vessels, and smooth muscle layers, controlling motility, secretion, blood flow, and nutrient absorption, in a rather autonomous way from the CNS [119]. However, these functions are not independently regulated by the ENS, since a complete control depends upon the integration of local reflexes, with reflexes mediated by intestinal fugal neurons synapsing with sympathetic ganglia, and by vagal, splanchnic, and pelvic afferent pathways to the CNS [90,119,120]. The human ENS is composed of a vast number of neurons, about 200–600 million neurons, the same number present in the spinal cord, with four major functional neuron types, intrinsic primary afferent neurons (IPANs), interneurons, excitatory and inhibitory motor neurons contributing to the formation of the subserous, myenteric and submucosal plexuses [119]. IPANs are sensory neurons detecting diverse stimuli (i.e. chemical, including microbial metabolites, and mechanical) within the mucosa and muscularis propria and initiating motor, secretory and vasomotor local reflex responses, but also, as recently proposed, more long reflexes involving the CNS [121]. Major features of neuroplasticity involving the ENS during inflammation encompass structural changes, ranging from nerve rearrangement (i.e., hypertrophy and hyperplasia) to degeneration and loss of enteric ganglion cells as well as altered neurotransmission, leading to gastrointestinal dysfunction characterized by sensory-motor and secretory impairment of the gut, which may also occur in segments of the gastrointestinal tract remote from the site of the original inflammation. Such neuroplastic changes result from a dynamic interplay between enteric neurons and different cell types within the enteric microenvironment, including immune cells, enteric glial cells, and commensal bacteria [7,8,9,122]. It is well established that the microbiota may influence both sensory and motor gut functions as well as the development of the ENS in critical postnatal life periods [123,124]. On the converse, the ENS by regulating the intestinal motor function may influence the microflora composition and load, allowing the elimination of redundant bacteria from the lumen [125]. The ability of the gut microbiota to influence ENS development may, at least in part, explain the increasing incidence of pediatric IBD, which especially involves those children previously exposed to antibiotic courses [126]. Interestingly, in a recent study, a “pro-inflammatory” microbial lineage developed in zebrafish carrying a genetic mutation that prevents the formation of the ENS (Sox10^−/−^). The inflammatory phenotype could be reduced by either transplanting wild-type ENS precursors or by administrating “anti-inflammatory” bacterial species [127]. Newborn GF mice display important ENS anomalies, which are not observed in the conventionalized ex-GF counterpart and specific pathogen free (SPF) controls [128,129]. In dysbiotic juvenile mice undergoing massive antibiotic treatment, complex morpho-functional neuromuscular rearrangements of the ENS were associated with up-regulation of brain-derived neurotrophic factor (BDNF) and its high-affinity receptor tropomyosin-related kinase B (TrkB) in myenteric IPANs [123,130]. Dysbiosis-induced neurochemical derangement in the juvenile mouse ENS involves activation of TLR2 and TLR4 [116,131]. Several studies resorting to TLR2 and TLR4 mutant mice suggest the importance of these receptors in maintaining ENS homeostasis, by influencing both neuronal and enteric glial function [130,131,132]. This latter observation is of particular importance given the fundamental role played by enteric glial cells in the modulation of neural circuits within the ENS. In this context, improper activation of signals on enteric glial cells, including those derived from bacteria, is suggested to underlie the development of neurodegenerative processes in IBD [7,132,133]. IPANs may be particularly sensitive to direct and indirect modulation by bacterial metabolites. In GF, mice myenteric IPANs displayed in vitro reduced excitability, which was restored after colonization with normal gut microbiota [134,135]. Interestingly, probiotics such as *Lactobacillus reuteri* may influence IPAN electrophysiological responses by increasing the excitability and the number of action potentials per depolarizing pulse, decreasing calcium and potassium channel opening and reducing slow after-hyperpolarization [134]. In this view, specific bacterial strains may be involved in maintaining intestinal neuromuscular function homeostasis. GF rats displayed a significant delay in the intestinal transit and in the migrating motor complexes (MMC) period, which were partially reversed after colonization with either *Lactobacillus acidophilus* or *Bifidobacterium bifidum,* while colonization with *E. coli* and *Micrococcus luteus* delayed gut motility [136]. The possibility to restore IPANs function by probiotic administration may be of interest in IBD since hyperexcitability of IPANs, facilitated fast synaptic transmission in both the submucosal and myenteric ganglia, and intestinal dysmotility represent persistent signs of inflammation-induced neuroplasticity, which may explain the continued dysmotility in patients with IBD in remission [8].

### 3.3. The Vagus Nerve

The vagus nerve has a fundamental role in microbiota–gut–brain communication. The vagus nerve with its 80% afferent and 20% efferent fibers innervates the mucosal and muscular layers of the entire gut from the esophagus to the distal part of the descending colon [137,138]. Vagal afferents within the gut epithelium serve to detect chemical stimuli, to regulate satiety, to allow food particle passage, to sense mucosal hormones, neuromodulators/neurotransmitters, or luminal molecules, such as bacterial metabolites to start neuronal reflexes [139]. Vagus nerve terminals in the epithelial layer are not normally exposed to stimuli originating from the gut luminal side and are indirectly activated by compounds released from enterochromaffin and enteroendocrine cells, such as serotonin (5-HT) via 5-HT_3_ receptors, cholecystokinin, histamine, and somatostatin [140,141]. TLRs and receptors for bacterial metabolites, such as SCFAs, are expressed on enteroendocrine cells, and their activation triggers vagal afferent excitation [93]. Furthermore, both SCFAs receptors and TLRs, such as TLR4, are expressed on vagal afferents, and these fibers can directly sense PAMPs, such as lipopolysaccharide (LPS), as well as SCFAs, such as butyrate [137,141], to activate the brain. LPS can also directly activate vagal afferent fibers in the NVG providing an explanation for the incomplete blockade of peripheral LPS or interleukin-1β-mediated effects on behavior after subdiaphragmatic vagotomy [142,143]. At a local level, the microbial flora is crucial for the normal activity of vagal afferent nerves, whose excitability was significantly reduced in GF mice versus their conventionalized ex-GF counterpart as well as to SPF animals [129]. Given the fundamental role played by vagal pathways in the control of gut motor and secretory functions, changes in the enteric microbiota composition and function may predispose the host to the motor and secretory dysfunctions, resulting in diarrhea or constipation. In turn, changes in vagus nerve activity may influence the enteric microbiota homeostasis, as suggested by bacterial overgrowth in the small intestine caused by compromised MMC motor complexes, which are under vagal control [144]. The existence of a reciprocal relationship between the vagus and the microbiota extends from the gut to the CNS along the microbiota–gut–brain communication axis [93]. Indeed, signals originating from the gut microbiota may reach the CNS via vagal pathways and vice-versa possibly favoring the development of altered brain responses. Several preclinical studies are available to demonstrate the involvement of vagal pathways in the modulation of microbiota-mediated stress-related disorders, such as anxiety and depression, both representing IBD neuropsychiatric symptoms [22,24]. In mice, the vagus nerve was shown to participate in microbiota-induced anxiety-related behaviors induced by oral administration of *Campylobacter jejuni* (*C.jejuni*) [145] and was associated with changes in c-Fos expression in NVG cell bodies and the NTS, suggesting the occurrence of rapid afferent signals after exposure to potential pathogens [146]. Analogously, in rats, deafferentation of the subdiaphragmatic vagus nerve contributed to the development of behavioral anomalies associated with altered gene expression in the NA [147]. Unlike endocrine and immune signals, this swift microbe-driven effect on the vagus may favor a rapid communication between the periphery and the brain, conceivably supporting a positive effect of probiotics on the CNS [148]. In mice, mesenteric nerve bundle firing increased after administration of *Lactobacillus rhamnosus* (JB-1) and was associated with changes in GABA expression in different brain regions as well as to reduced stress-induced corticosterone anxiety and depression-related behavior, which were dependent on an intact vagus nerve [149,150]. Interestingly, in a mouse model of colitis induced by oral administration of DSS, the probiotic *Bifidobacterium longum* reduced the anxiolytic effects associated with the development of gut inflammation, and this action required vagal integrity [151]. However, non-vagal pathways are also involved in the protective effects of *Lactobacillus reuteri* and *Bifidobacterium infantis* after DSS-induced colitis in mice, since both probiotics were effective even after subdiaphragmatic vagotomy [152]. The vagus nerve is described to have anti-inflammatory properties via the so called “vago-vagal anti-inflammatory reflex loop” also defined as the “cholinergic anti-inflammatory pathway” [93]. In this context, peripheral stimulation of vagal afferents by pro-inflammatory cytokines released by the intestinal mucosa (IL-1β, IL-6, TNF-α) activates vagal efferents to peripherally release acetylcholine, with the consequent inhibition of TNFα release by macrophages through α7nicotinic cholinergic receptors [153,154]. Overexpression of TNFα represents a well-documented mechanism in the development of IBD [155] and the ability of the vago-vagal reflex to inhibit pro-inflammatory macrophages may ameliorate intestinal permeability and influence gut microbiota homeostasis during inflammation [156]. In support of this hypothesis, vagus nerve stimulation has been shown to reduce systemic inflammatory responses to endotoxin and intestinal inflammation and to indirectly modulate spleen immune activity by interacting with the splenic sympathetic nerve [154,157,158]. Accordingly, in rats, chronic vagus nerve stimulation after trinitrobenzene sulfonic (TNBS) acid-induced colitis improved the disease index and led to a reduction in inflammatory markers [159]. In vagotomized mice a more severe form of inflammation developed after DSS-induced colitis, which was associated with increased levels of IL-1β, IL6, TNFα, and NF-κB levels [160,161]. The “cholinergic anti-inflammatory vagal pathway” has been suggested to participate in the development of a depressive mood associated with anxiety in IBD, since mice developing depressive-like behavior after reserpine treatment, which reduces intestinal acetylcholine levels, present more severe DSS- and DNBS-induced colitis [162]. Interestingly, a reduction of the vagal tone has been observed both in both IBS and IBD patients characterized by increased epithelial permeability and dysbiosis [163,164]. In the reciprocal interplay between the host and the microbiota, the saprophytic microflora by modulating vagal signaling to the CNS may influence vagal inflammatory reflex, underlying anti- or proinflammatory effects [93,153]. Thus, monitoring the vagal tone as a marker of the microbiota–gut–brain axis function may represent a promising approach to elucidate the complex IBD pathophysiology and possible microbiota–gut–brain axis-based therapies.

### 3.4. Spinal Pathways and Visceral Pain

Changes in gut–brain axis communication may contribute to the development of chronic visceral pain and hypersensitivity, representing the most common cause of disability and impaired quality of life in IBD [165,166]. Up to 70% of IBD patients refer of abdominal pain symptoms, more frequently reported in pediatric than adult patients, and about 20–60% of IBD patients experience persistent pain during remission [167,168,169]. Symptoms of abdominal pain may persist after successful treatment of the active disease, sustaining IBS-like symptoms, which raise in approximately 40% of IBD patients, and with a higher prevalence in CD than in UC patients [165]. Pain sensing neurons are represented by a specialized population of primary afferent neurons, called nociceptors, whose cell bodies reside in dorsal root ganglia (DRG) and represent polymodal C fibers that relay a variety of potentially noxious stimuli (mechanical, chemical, and thermal) to the CNS, where the pain signal is elaborated [90,170]. Sensitization of primary sensory afferents innervating the viscera, hyperexcitability of ascending spinal neurons (central sensitization) receiving synaptic stimuli from the gut, and dysregulation of descending pathways modulating spinal nociception represent the main mechanisms underlying the development of chronic visceral pain and hypersensitivity [167,171,172,173]. In this latter context, reports are suggesting that permanent alterations in stress-responsive regions and the descending pain modulatory system are involved in chronic visceral hypersensitivity associated with gut inflammatory conditions [171]. Neuroplasticity changes in both extrinsic primary afferents and IPANs occur in response to alterations of the enteric microenvironment as a consequence of increased epithelial permeability, enhanced interactions with the saprophytic flora, and pathogen microorganism, increased neuroimmune interplay and gliosis, which contribute to enhancing visceral nociception and gastrointestinal dysfunction [8,90,170]. The involvement of the gut microbiota in the modulation of visceral pain and hyperalgesia has attracted much attention in the last decades [165,174]. In GF mice the excitability of myenteric IPANs decreased versus SPF controls and was restored after colonization, suggesting that a normal intestinal microbiota is essential for their activity [175]. The extrinsic sensory innervation is also sensitive to the composition of the microflora since, in mice, administration of live *Lactobacillus reuteri* (DSM 17938) reduced jejunal spinal nerve firing evoked by gut distension with an intraluminal balloon or capsaicin [176]. Antibiotic treatment in rat pups induced visceral hypersensitivity in adult male rats and was associated with a decrease in the transient receptor potential vanilloid member 1 (TRPV1), the alpha-2A adrenergic receptor, and cholecystokinin B receptor expression in the spinal cord, while probiotic treatment ameliorated these symptoms [177,178]. These observations are strongly indicative for the existence of a correlation between dysbiosis occurring in early-life and the development of visceral pain in adulthood [179]. Administration of the probiotic *Bifidobacterium infantis* 35,624 ameliorated visceral hypersensitivity in a mouse model of TNBS-induced colitis while *Lactobacillus paracasei* NCC2461 and *Lactobacillus reuteri* reduced nociceptive responses giving direct evidence for a role of the microbial flora in the regulation of visceral pain associated with chronic intestinal inflammation [178,180,181,182].

Visceral hypersensitivity in GF mice is associated with an increase in TLRs receptors and pro-inflammatory cytokine levels such as IL6 or TNFα [183,184]. The release of several mediators of inflammation, such as neuropeptides, cytokines, and prostanoids induced by low-grade inflammation, has been suggested as a possible mechanism underlying stimulation and sensitization of sensory afferent nerve endings, triggering visceral hypersensitivity [185]. Elevated levels of TNFα were found in colonic biopsy samples from patients with active UC, and supernatants from these biopsies could activate and sensitize colonic DRGs via the TNFα receptor, TNFR1, suggesting a possible pathogenetic mechanism for altered visceral pain perception in patients with active UC [186]. Despite this evidence, the effect of microbiota manipulation on IBD-associated visceral hypersensitivity still remains to be largely elucidated, and only a few clinical studies have been carried out to evaluate the possible beneficial effects with variable outcomes, as pointed out in successive paragraphs of this review.

### 3.5. Hormonal Connections: The HPA Axis and Stress Response

Along the bidirectional gut–brain communication axis, the neuroendocrine hypothalamic-pituitary-adrenal (HPA) axis regulates several functions, comprising visceral sensation and stress responses at a central level, and epithelial permeability in the gut [22,24]. The activated HPA axis causes the secretion of corticotropin-releasing factor (CRF) from the paraventricular nucleus (PVN) of the hypothalamus, which stimulates the pituitary gland to release the adrenocorticotropic hormone (ACTH). In turn, ACTH triggers the release of the immunosuppressive stress-hormone cortisol from the adrenal cortex. Several clinical studies suggest that among the environmental factors contributing to IBD pathogenesis, psychosocial stress has a prominent role, with the majority of IBD patients referring to a correlation between the level of stress and the degree of symptom severity [187]. Data from preclinical investigations suggest that psychosocial stress can reactivate gut inflammation during experimental colitis in laboratory animals [188]. Furthermore, in the clinical context, adverse life events and emotional conflicts, often associated with anxiety and depression, represent causal factors for the exacerbation of symptoms as well as of IBD relapses [24,189]. A recent study based on the integration of 3D genomic data with publicly available GWAS data for depression and IBD traits to identify genetic commonalities is highly suggestive for the existence of potential genetic relationships between IBD and stress-induced depression, involving key stress regulator hypothalamic genes [190]. In IBD, stress-induced brain–gut perturbations are associated with mast cell-mediated pro-inflammatory responses in the periphery and the CNS and with a complex array of neuroendocrine and autonomic reflexes consequent to HPA axis activation, increased pro-inflammatory sympathetic outflow, and decreased anti-inflammatory vagal outflow, mainly under the control of the prefrontal cortex and amygdala [24]. The microbiota represents a further player in the modulation of stress responses, and the ability of gut microbes to control the development and function of the HPA axis has been demonstrated in different preclinical studies, mainly resorting to GF animals, after antibiotic or probiotic treatment [123,191,192]. In a seminal study on GF mice, a mild stress restraint increased corticosterone and ACTH plasma levels, and this effect was reversed by specific colonization with *Bifidobacteria* species [191]. These observations have been confirmed by successive preclinical studies showing that probiotic treatment may normalize HPA axis dysfunction induced by stress in early-life, which represents an important risk factor for the development of colitis later in life [192,193]. Prevention of gut epithelial barrier impairment by a probiotic treatment led to attenuated HPA response to acute psychological stress in rats [194]. A bidirectional microbial-neuroendocrine relationship seems, however, to exist, since stress may have long-term effects on the microbiota composition both in early-life and in adulthood [195,196]. Stress-induced cortisol secretion can affect immune cells and cytokine release in the gut, altering the epithelial barrier permeability and function and, consequently, the gut microbiota homeostasis and composition [197,198,199]. Indeed, prolonged exposure to stress induces ultrastructural changes of the intestinal barrier, favoring the systemic translocation of different bacterial strains, such as *Lactobacillus* spp. and activation of an immune response [200,201]. Activation of the innate immune system sustains the development of a proinflammatory state and secretion of intestinal secretory IgA, eventually reinforcing dysbiosis [202]. Besides, neuroendocrine/neurotransmitter signals generated from stress responses, such as catecholamines, may facilitate bacterial growth, as suggested for non-pathogenic *E. coli* as well as for pathogenic strains, such as *E. coli* 0157:H7 [203]. The consequences of stress-induced dysbiosis were demonstrated also in the CNS with the development of neuro-immune responses, involving TLR-mediated neuroinflammation, which was prevented by antibiotic treatment [204]. Perturbations of the microbiota–gut–brain axis may have important consequences on the expression and activity of glutamatergic NMDA receptors and the related brain-derived neurotrophic factor (BDNF), both fundamental for neuroplasticity in brain regions, such as the hippocampus, amygdala, and cingulate cortex, all involved in stress responses [26,123,205]. Data obtained from preclinical studies suggest that stress-related mood disorders, such as major depression and generalized anxiety, commonly diagnosed in IBD patients may be related to alterations in the saprophytic microflora homeostasis [15]. In dextran sodium sulfate (DSS)-treated mice, colitis was associated with anxiety behaviors and cognitive deficits, which were prevented by a probiotic (*Lactobacillus rhamnosus R0011* and *Lactobacillus helveticus R0052*), suggesting the involvement of the gut microbiota in behavioral disturbances associated with intestinal inflammation [206]. Interestingly, DSS-induced colitis in weaning mice induced cognitive deficits and anxiety-like behaviors in conjunction with neuroinflammation and decreased neurogenesis in the hippocampus, as well as dysbiosis with reduced butyrate-producing species, in adulthood. Overall these observations strengthen the concept that colitis-induced changes in microbiota–gut–brain axis in early-life may persist in adult age [207]. Ampicillin exposure caused anxiety and colitis in mice, promoting monocyte/macrophage-activated gut inflammation and neuroinflammation in the cortex and hippocampus, which were associated with elevated levels of Proteobacteria, and were reversed by Lactobacilli administration [208]. Recently, increased TNF-α and IL-6 expression in the rectum and hippocampus activated caspase-3 in the hippocampus and decreased hippocampal neurogenesis, associated to colitis and development of depressive-like behavior in mice after DSS-treatment could be reversed after a long-term treatment with *Enterococcus faecalis* 2001 [209]. In rats, after experimentally-induced colitis, anxiety- and depression-like behaviors were associated with enhancement of colonic afferent firing rates and could be recorded only with intact vagal afferents, indicating that these behavioral changes were mainly of neuronal origin [151]. However, in another study, the same group showed that experimentally-induced inflammation in mice by administration of the non-invasive parasite, *Trichuris muris*, induced psychological disturbances probably via non-neuronal, immune pathways since anxiety-like behaviors were not prevented by vagotomy [210]. Although much of the current knowledge on the relationship between the gut microbiota, stress, and stress-related mood disorders in IBD relies on animal studies, emerging clinical evidence on the beneficial effect of microbiota manipulation in the treatment of other stress-related conditions lent support to the development of studies aiming at discovering novel biomarkers and target adjuvant therapies for IBD in the field of “psychobiotics” [22].

## 4. Microbial Metabolites and IBD

**Figure 2:**
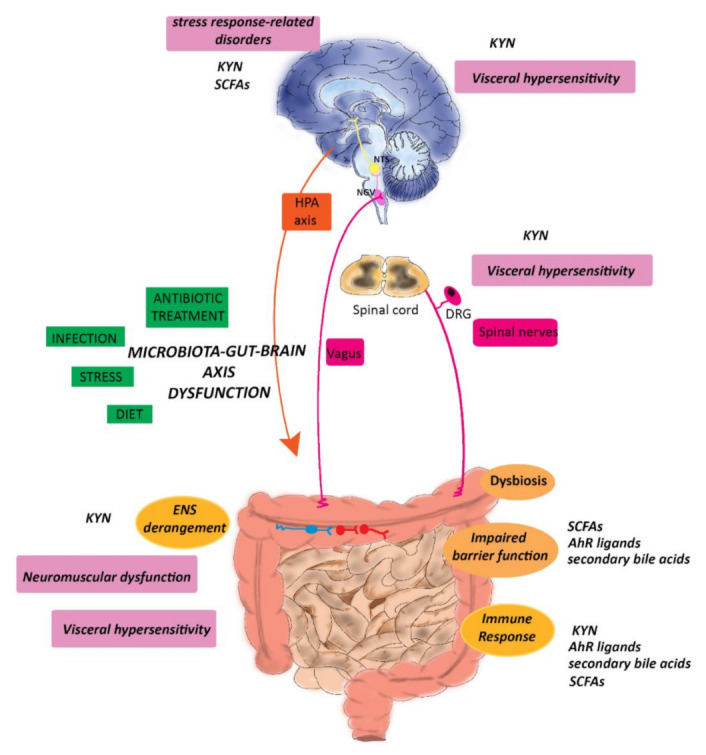
Potential role of microbial metabolites in the modulation of symptoms associated with IBD. Environmental changes, stress, diet, previous infection, and dysbiosis may alter the homeostasis of the gut–brain axis underlying the development of motor dysfunction, visceral pain, and stress-related responses, such as anxiety and depression, in IBD patients. Tryptophan metabolites, SCFAs, and secondary bile acids by influencing the epithelial barrier and the immune function and KYN by modulating the ENS peripherally influence symptom development, as described in the text. Furthermore, changes in KYN and SCFAs brain levels may underlay the development of mood disorders and anxiety, while KYN may be involved in visceral hypersensitivity by modulating visceral pain along the gut–brain axis. Abbreviations: DRG, dorsal root ganglion; KYN, kynurenine; IBD, Inflammatory Bowel Disease; NGV, nodose vagal ganglion; NTS, the nucleus of the solitary tract; ENS, enteric nervous system, HPA, hypothalamic–pituitary axis, SCFAs, short-chain fatty acids (with modifications from Baj et al., 2019 [26]).

The symbiotic relationship between the gut microbiota and the host is the result of a dynamic and beneficial equilibrium between both players, and exponentially growing studies focus on the fascinating interkingdom of communication pathways developed between prokaryotic and eukaryotic cells in health and disease conditions Figure 2. Microorganisms harboring the human gastrointestinal tract produce many pleiotropic compounds, such as vitamins, gas, organic acids, bile salts, and bacteriocin, influencing in the host innate and acquired immunity maturation and homeostasis, energy, and metabolism and maintenance of the epithelial barrier function, providing defense against pathogens [26]. GF animals display significant differences in metabolite levels in different biological tissues including the gut, if compared with conventionally reared controls, necessitating a higher caloric intake to maintain the same body weight and are prone to vitamin deficiencies requiring dietary supplementation [211,212]. Dysbiosis associated with IBD may alter the bacterial metabolic profile influencing the host organism homeostasis with prominent alterations in the levels of metabolites with immunomodulatory properties, such as SCFAs, bile acids, and tryptophan metabolites predisposing to mucosal inflammation [213]. Defects in the production of protective bacterial metabolites may adversely influence gut–brain communication favoring gut–brain disorders associated with IBD. (Figure 2).

### 4.1. Short-Chain Fatty Acids

In the gut SCFAs, mainly as acetate, propionate, and butyrate, represent the principal products derived from the fermentation of dietary fibers induced by commensal microbes, as indicated by the drastically lower levels of these metabolites in both GF and dysbiotic animals versus their counterpart controls [214,215]. SCFAs are produced in the colon, mainly by *Firmicutes* and *Bacteroidetes*, in a molar ratio of 60:20:20 for acetate, propionate, and butyrate, respectively, and are rapidly absorbed by colonocytes, representing an energy source for these cells [216]. SCFAs improve and regulate the intestinal barrier integrity, water absorption and mucous production, and gastrointestinal motility. Several in vitro and in vivo preclinical studies have shown that SCFAs may modify colonic motor patterns by promoting the release of neuroendocrine modulators from the epithelium, such as GLP-1 and PYY from enteroendocrine L cells, and 5-HT from enterochromaffin [217,218]. When transported in the systemic circulation SCFAs can interact with different immune, endocrine, neuronal, and humoral mechanisms, influencing the host gut–brain signaling pathways, thus having a central role in the maintenance of brain homeostasis and function [219,220,221]. SCFAs may accomplish their modulatory functions by acting as epigenetic modulators of gene expression, promoting histone hyperacetylation through the inhibition of histone deacetylases (HDACs), and as endogenous ligands for several G protein-coupled receptors (GPCRs), such as free fatty acid receptor 2 (FFAR2) and 3 (FFAR3) and GPR109A [222,223,224]. Expression of these receptors has been reported in several peripheral organs, including the gastrointestinal tract, and in the CNS [225,226]. SCFAs are among the most widely studied microbial metabolites in IBD [213]. Both SCFA receptors and epigenetic mechanisms are involved in gut immune responses by regulating the differentiation, recruitment, and activation of neutrophils, macrophages, dendritic cells, and monocytes [225]. In rodent models, oral administration of butyrate and propionate promoted peripheral Treg cell generation [226] (Table 2). In murine models of IBD, specific bacterial strains producing SCFAs were shown to increase the function of mucosal Treg, promoting tolerance, and reducing inflammation [227,228]. Analogously, in the mouse colon lamina propria, butyrate has been shown to modulate macrophage activity by inhibiting the transcription of proinflammatory molecules, providing a status of tolerance toward microbial commensals [229]. In mice, susceptibility to DSS-induced colitis was reduced by very high dietary fiber intake or by SCFAs, via FFAR2 and GPR109A receptor activation and the downstream cascade of NLPR3-inflammasome IL-18 axis [230]. These preclinical observations are of translational value since it is now clear that IBD may correlate with impaired SCFAs-fermentative pathways, decreased levels of SCFAs-producing bacteria, and lower amounts of fecal SCFAs [81,207]. In this context, it is proposed that SCFAs production is linked to a reduced IBD risk [231]. Indeed, IBD patients show reduced lower steady state levels of SCFAs in intestinal mucosa and feces, matching with the significant reduction of dominant SCFAs-producing bacteria, such as *Faecalibacterium prausnitzii* and *Roseburia intestinalis* [81,82,231]. Furthermore, in IBD patients, a significant correlation has been suggested to exist between the degree of inflammation and inhibition of genes related to SCFAs uptake and metabolism [232]. The expression of the butyrate transporter MCT1 and its gene, SLC16A1, is reduced in inflamed mucosa of UC and CD patients [232,233]. Analogously, genes encoding enzymes catalyzing butyrate metabolism/oxidation (such as ACSM3, ACADS, ECHS1, HSD17B10, ACAT1, ACAT2, ABAT, ALDH1A1, ALDH2, ALDH9A1, EHHADH, HADHA, HMGCL, and PDHA1) are down-regulated in inflamed mucosa of UC patients, suggesting that inflammation is tightly linked to the inhibition of host genes related to SCFAs uptake and metabolism [232,233,234]. Given the important contribution of SCFAs to gut–brain signaling, changes in their levels during IBD may have a role in the appearance of brain disorders associated with the disease [11,28]. Circulating SCFAs can cross the blood–brain barrier (BBB) to a minimum extent, albeit exerting neuroprotective properties [220]. Injection of butyrate in rat brains enhanced mRNA levels of neurotrophic factors, such as BDNF and GDNF, improving neurogenesis, synaptic plasticity, memory formation, and mood-related behaviors [220]. Similarly, in the mouse hippocampus after pneumococcal meningitis, butyrate prevented memory impairment by reestablishing BDNF and GDNF levels [235]. In mice brain, acetate, propionate, and butyrate may also influence levels of neurotransmitters, such as catecholamines, glutamate, and GABA, these latter playing a primary role in the modulation of behavior disorders, such as anxiety and depression [26,236,237]. Interestingly, in mice, chronic administration of butyrate and the consequent short-lasting histone hyperacetylation in the hippocampus and frontal cortex exerted an antidepressant-like effect, inducing a significant decrease of behavioral despair [238]. SCFAs may indirectly modulate mood and behavior by promoting the release of neuropeptides from colonic enteroendocrine cells, such as GLP1 and PYY, which are involved in the processes of learning and memory and the modulation of affective states [239,240]. Evidence from preclinical data suggests that SCFAs are also involved in gut inflammation-induced stress responses. In mice, alterations of SCFAs, their receptors, and *Lactobacillus spp* levels producing SCFAs have been demonstrated after stressor restraint during infection with *Citrobacter Rodentium* underlying gut inflammation [241]. Accordingly, the supplementation of butyrate, acetate, and propionate in drinking water can ameliorate reward-seeking behaviors and the response to stress in mice [242]. The gut microflora may influence stress-responses and behavioral disorders via SCFA-mediated modulation of vagal and HPA axis [242,243]. Experimental studies resorting to the use of prebiotic, probiotic, and dietary to demonstrate SCFA-mediated modulation of stress reactivity, affective and cognitive processes, and behavior have been prevalently carried out resorting to animal models and to a less extent in humans with contradictory results [221]. However, in a recent triple-blind, randomized, placebo-controlled intervention trial, administration of low and high doses of SCFAs to healthy volunteers significantly attenuated the cortisol response to psychosocial stress compared to placebo suggesting that colon-delivered SCFAs modulate HPA axis reactivity to psychosocial stress [244].

### 4.2. Bile Acids

The gut commensal flora mediates the de-conjugation and de-hydroxylation of conjugated primary bile synthesized from cholesterol in the host liver, into secondary forms with pleiotropic functions, comprising regulation of systemic lipid, cholesterol, and glucose metabolism and the immune function [245]. Bile acids exert their actions via nuclear hormone farnesoid X receptor (FXR) and the G protein-coupled bile acid receptor 1 (TGR5) activation. Different studies have shown that Trg5^−/−^ deficient mice have decreased bile acid pools [246], and targeted deletion of intestinal Fxr protects mice against liver steatosis and obesity, when fed with a high-fat diet [247,248]. Bile acids and gut microbiota are in constant bidirectional communication, influencing each other’s composition [27]. Deficiency in luminal bile acids favors small intestine bacterial overgrowth, activation of inflammation, and gut epithelium damage, suggesting an important role played by these metabolites and their receptors as components of intestinal antimicrobial defense [249,250]. In the small intestine, FXR is involved in the regulation of antimicrobial defense gene expression, such as iNOS and IL-18, warranting protection from epithelial damage and bacterial translocation [249,251]. In this context, secondary bile acids are suggested to play an important role in the modulation of gut inflammatory responses, as also suggested by their decreased fecal and serum content in IBD patients [252]. Several studies have demonstrated that FXR activation is correlated with reduced severity of experimentally-induced colitis in mice and inhibition of proinflammatory cytokine production in lamina propria mononuclear cells from IBD patients [253,254]. Single nucleotide variations in FXR have been investigated to evaluate possible links to IBD susceptibility, although, with low degrees of success [255,256]. In a recent retrospective cohort study, however, a higher risk of developing CD progressing on to surgery was observed in women carrying the FXR-1GT gene polymorphism, providing a putative useful screening tool to identify female patients at risk for poor disease outcomes [257]. TGR5 is also directly involved in the modulation the gut-associated lymphoid tissue and in the control of gut inflammation [258,259]. Trg5^−/−^ mice display an increased intestinal permeability versus their controls [258], and the addition of several TGR5 activating compounds leads to attenuated production of pro-inflammatory cytokines and inflammatory processes in mouse models of colitis [260,261,262]. In the context of host-microbiota interplay, secondary bile acids have been shown to ameliorate mouse experimental colitis by favoring the expansion of anti-inflammatory cluster XIVa *Clostridium* and *Akkermansia muciniphila* [263]. Maintenance of epithelial integrity impacts gut–brain communication and the hypothesis that secondary bile acids may participate in microbiota–gut–brain communication by modulating inflammatory pathways during IBD is intriguing [25]. Indeed, the blood–brain barrier is permeable to secondary biliary acid of gut microbial origin, and their receptors have been found in the brain [264]. A correlation between deficient bile acid deconjugation and impairment of both intestinal barrier function and behavior was found in a transgenic mouse model of autism spectrum disorder [265]. This evidence was further supported by the reduction in the relative abundance of particular bile-metabolizing bacterial taxa such as *Bifidobacterium* and *Blautia* species. Furthermore, several data, mainly from preclinical studies, suggest that bile acid may have a neuroprotective role in different neurological diseases, including Alzheimer’s, Parkinson’s, Huntington Disease, all characterized by a prominent degree of microgliosis and neuroinflammation [264]. However, the possibility that secondary bile acids may represent a direct molecular link between the gut microflora and the brain remains largely unexplored, and no data are available at the moment, at least up to our knowledge, on their involvement with the development of IBD psychiatric symptoms [264].

### 4.3. Trp Metabolism: Focus on Kynurenine and AhR Ligands in IBD

Several reports are available to suggest that changes in L-tryptophan (TRP) metabolism may have a role in the development of inflammatory responses in the gut [118]. This essential amino acid is fundamental for human health, representing the precursor of important bioactive molecules, some of which are modulators of gut–brain axis communication pathways [118,266]. TRP cannot be synthesized by the human body, and it is mainly obtained from nutritional diet sources [267]. In the gut, TRP undergoes two major metabolic host pathways, the kynurenine (KYN) and serotonin (5-HT) biosynthetic pathways, and one microbial pathway to produce indole and its derivatives [118]. The highest amount of the absorbed TRP (about 90%) is metabolized by the enzymes indoleamine-2,3-dioxygenase (IDO1) and tryptophan-2,3-dioxygenase (TDO) along the KYN biosynthetic pathway [268], and approximately 3% is transformed into serotonin 5-HT via tryptophan hydroxylase 1 (TPH1); the rest is degraded by the gut microflora [269]. In IBD, especially in CD patients, TRP metabolism increases; consequently, the amino acid levels are reduced with respect to normal healthy individuals, and these changes correlate with the gravity of the disease [270,271,272]. Interestingly, mice undergoing a TRP-free diet developed a more severe and rapid inflammatory response to DSS [273]. During active IBD, decreased TRP serum levels are associated with unbalanced amino acid metabolism, shifting towards the KYN arm [270,272].

#### 4.3.1. Kynurenine

KYN pathway biosynthetic enzymes, IDO-1 and TDO have a tissue-dependent distribution, since IDO-1 is localized in most tissues including the intestine, and TDO is mostly expressed in the liver and responsible for the majority (almost 99%) of KYN production [266]. Both enzymes have also distinct functions, since IDO represents the rate-limiting step of KYN formation and is activated by proinflammatory cytokines, while TDO is regulated by glucocorticoids [274,275].

Both host’s KYN biosynthetic pathways are modulated by the commensal microflora [276,277]. Changes in IDO and TDO activities in GF animals and dysbiotic animals were associated with increased circulating TRP levels and a consequent decrease of the KYN-to-TRP ratio [278,279,280,281]. Such an unbalanced ratio could be restored by the administration of probiotics such as *Bifidobacterium infantis* [281]. The KYN arm of TRP metabolism has important physiologic functions both in the periphery and in the CNS, via downstream by-products such as kynurenic acid (KynA) and quinolinic acid (QA) and, as more recently demonstrated, picolinic acid (PA) [266,267,282]. As part of an interkingdom bidirectional communication system, activation of TLR-3 on monocytes may enhance KYN downstream production of both KynA and QA [283]. From a mechanistic viewpoint, QA is an agonist at the NMDA glutamate receptor and is considered as a neurotoxic pro-inflammatory molecule [284], while KynA is a natural competitive antagonist for the glycine site associated with NMDA receptor, α7nACh, and GPR35 receptors [91,267]. KynA protects neurons against glutamate-mediated excitotoxicity and is considered neuroprotective both in the CNS and in the ENS [285,286,287]. QA, KynA, and PA have also immunomodulating properties and can influence the gut immune function and inflammatory responses [288,289]. Alterations of cytokine, cortisol, and microbiota level and composition during gut inflammation favor IDO activation and the consequent enhancement of KYN levels [290,291,292]. Increased KynA levels were found in the serum and biopsies of IBD patients [293,294]. Upregulation of IDO1 is a common hallmark to IBD and metabolic disorders, such as obesity and insulin resistance, all characterized by low-grade, Th1-mediated inflammation. In this context, activation of the KYN pathway may have an immunomodulatory function by reducing Th1 responses and enhancing Th2-mediated processes [295,296]. Indeed, several reports suggest that KynA has anti-inflammatory properties by modulating immune cells differentiation and function, blunting proinflammatory cytokine levels as well as favoring the expression and secretion of anti-inflammatory and protective factors such as α-defensine and TSG-6 [297,298,299]. As an anti-inflammatory agent, KynA also inhibits xantine oxydase in vitro, resulting in reduced production of oxygen species [300]. These positive, beneficial effects may, however, be reduced by the increased release of toxic metabolites such as, QA [295]. In the gut neuromuscular compartment, the modulation of the KYN pathway towards KynA synthesis is suggested to be neuroprotective to compensate for inflammation-induced NMDA receptor activation [91]. Enteric NMDA receptors have neuroinflammatory properties by sustaining oxidative and nitrosative stress responses [301]. Interestingly, in TNBS-treated rats, administration of KynA and its synthetic analogue SZR-72 reduced nitrosative stress and IL-6 and TNFα release and ameliorated motility patterns suggesting the involvement of enteric NMDA receptor pathways [302]. In this context, both in preclinical and human studies, KynA and its derivative have been considered as potential therapeutic tools for IBD management [301].

The increased ratio KYN-TRP and the consequent diversion of TRP metabolism from the 5-HT pathway towards the KYN pathway, favored by changes in microbial composition, may be relevant also to the manifestation of IBD-associated mood disorders, such as anxiety and depression [26,118,275]. Increased anxiety-like behaviors developed in GF mice receiving gut microbiota from depressed patients in parallel with increased KYN levels and increased KYN/TRP ratio in blood circulation [303]. Accordingly, the conversion of TRP into KYN after INF-γ-mediated activation of IDO1 promoted the manifestation of depressive symptoms in chronic hepatitis C patients, free of psychiatric disorders [304], while administration of *Bifidobacterium infants* ameliorated the depressive behavior in rats subjected to the forced-swim test, which was correlated with normalization of TRP metabolism [281]. In mice administration of the non-invasive parasite, *Trichuris muris* induced experimental colitis and psychological disturbances, associated with enhanced KYN circulating levels and enhanced KYN/TRP ratio [210]. KYN levels and behavior were normalized after administration of classic anti-inflammatory drugs, such as etanercept and budesonide, used for IBD treatment [210]. Increased inflammation-induced KYN circulating levels may cross the BBB and translocate into the brain, where KYN is transformed into its metabolites, principally KynA and QA acid [275]. Microglia is the main cellular source for the neurotoxic QA, which is proposed to participate in depression development, while astrocytes produce KynA which displays some neuroprotection [305]. Data from clinical studies suggest that a high KynA/QA ratio may represent an index of neuroprotection, whilst a low ratio sustains the development of inflammation-induced depressive disorders [306]. An integral saprophytic microflora is suggested to modulate KYN homeostasis not only in the periphery but also in the CNS by influencing the maturation and function of microglia and astrocytes, indicating that modulation of TRP metabolism may represent a unifying mechanism linking inflammation-induced depression and dysbiosis along the microbiota–gut–brain axis [275]. An issue that needs to be explored is whether KYN derivatives may represent a possible novel signaling pathways to target visceral hyperalgesia in IBD, as suggested for IBS [307]. KynA by modulating NMDA receptor pathways may influence mechanosensitive pathways, transducing sensory stimuli deriving from pelvic and splanchnic afferents in response to neuroinflammation and hyperalgesia [91,122,308]. NMDA receptors located on complex neuronal networks in the spinal cord may promote the amplification of nociceptive signals and the “wind-up” of central responses to nociceptive stimuli [90,309]. Indeed, modulation of glutamatergic transmission along the gut–brain axis may participate in the adaptation of afferent neurons and CNS pain processing, leading to chronic visceral hypersensitivity in IBS patients [309]. In this context, the possibility that KynA and its synthetic analogues may offer novel therapeutic options devoid of significant adverse effects for the treatment of visceral pain associated with IBS and IBD, as already suggested for neuropathic pain syndromes, is intriguing [310,311].

#### 4.3.2. AhR Ligands

A fraction of intestinal TRP locally serves as a substrate for the production of protective metabolites consisting of indole and its derivatives, such as indole-3-aldehyde (IAld), indole-3-acetic acid (IAA), indole-3-propionic acid (IPA), indole-3-acetaldehyde (IAAld), and indoleacrylic acid [312]. Despite of the limited number of reliable methods for the determination of microbial TRP catabolites in human biological specimens, available data suggest that the indole, IAA, and IPA are the most abundant microbial TRP catabolites in the human body [313,314]. Indolic compounds are inter-species and inter-kingdom signaling molecules, which regulate different aspects of the bacterial physiology, such as sporulation, biofilm formation, and antibiotic resistance, and may support the host gut mucosal barrier and immune functions [282]. Indolic compounds are produced by the enzyme tryptophanase, which is expressed in a large number of microorganisms, including *E. coli*, *Proteus vulgaris*, *Paracolobactrum coliforme*, and *Achromobacter liquefaciens* [315]. Decreased indole levels during dysbiosis are associated with altered immune and epithelial barrier functions [316,317]. Indolic compounds by activating the cytosolic aryl hydrocarbon receptor AhR, a transcription factor, may support gut immune responses [318]. AhRs have been detected on Th17 cells [319], innate lymphoid cells [320,321], macrophages [322], dendritic cells [323,324] and neutrophils [325]. IAld, for example, favors IL-22 production and intraepithelial lymphocytes recruitment, via AhR activation [325,326]. In mice, *Lactobacillus* Spp., by influencing IL-22 production via IAld-mediated AhR activation, provided protection against mucosal candidiasis [327]. Noteworthy, indole derivative-mediated AhR activation can affect the differentiation of naive CD4^+^ T helper cells into Treg and Th17 cells, driving towards protective, anti-inflammatory responses [312]. An increasing number of studies suggest that AhR has a protective role during intestinal inflammation, and both preclinical and clinical studies suggest that IBD is associated with AhR downregulation [312,328,329]. Pharmacological manipulation of AhR expressed on mononuclear cells isolated from the intestinal mucosa of IBD patients induced up-regulation of IL-22 and downregulation of IFNγ, a pro-inflammatory cytokine [329]. In preclinical models of colitis and IBD patients, changes of AhR expression are associated with alterations of the serum and fecal levels of several AhR ligands [282,312]. Activation of AhR in a humanized murine model of IBD, whereby human CD4^+^ T cells induce colitis upon exposure to TNBS, reduced the inflammatory response by inducing Treg cells and favoring oral immune tolerance [330]. After DSS-induced colitis in caspase recruitment domain-containing protein 9 (Card9) knockout mice dysbiosis was associated with reduced IAA levels, reduced IL-22 production, and higher susceptibility to inflammation [331]. CARD9 is a susceptibility gene for IBD that modulates the immune response against microorganisms and promotes recovery from colitis by inducing IL-22 production. In the same study, reduced production of AhR ligands was also observed in the microbiota from individuals with IBD, particularly in those with CARD9 risk alleles associated with IBD, suggesting that host genes may alter the production of microbial metabolites during inflammation [331]. In a metabolomic study, selective diminution of IPA serum levels was shown in patients with active colitis versus healthy subjects [332]. A beneficial effect on chemically-induced inflammatory injury in mouse small and large intestine was obtained after oral administration of indole and IPA [332,333]. In vitro exposure of human intestinal epithelial cells to indole metabolites induced the overexpression of IL-10 receptor ligand-binding subunit (IL-10R1), blunting the production of proinflammatory mediators, thus sustaining the barrier homeostasis [332]. Strains of *Lactobacillus*, with AhR activating properties, reduced the severity of DSS-induced colitis in mice [331,334]. IA producing *Peptostreptococcus russellii,* carrying the *fld*AIBC phenyllactate gene cluster, reduced the vulnerability to colitis by improving goblet cell differentiation and reducing inflammatory signals [335]. These data reflect the ability of bacteria with mucin- and TRP-metabolizing abilities to ameliorate epithelial integrity, and it led the authors to identify a reduced abundance of phenyllactate gene cluster in UC by metagenomic analysis. Overall, these observations suggest that TRP metabolism underlays IBD pathogenesis and that changes in the microbiota composition may contribute to disease development, either by influencing AhR ligand levels or by modulating the host IDO and TPH1 activity. In this scenario, manipulation of TRP metabolism either via conventional pharmacological approaches or by the administration of pre- and probiotics targeting TRP metabolite-producing bacteria may represent promising novel therapeutic approaches in IBD patients.

## 5. Microbiota-Based Approaches in IBD Therapy

### 5.1. Antibiotics

Antibiotics may influence the course of IBD by decreasing concentrations of bacteria in the gut lumen and by modifying the gut microbiota composition. Moreover, antibiotics reduce tissue invasion, attachment, and translocation; may cause a shift in microbial metabolism, with an increase in SCFAs; and decrease bacterial enzyme activities, which correlates with the clinical response [337]. Recent data show that antibiotics may have direct effects on the host immunomodulatory functions by antagonizing the action of TNFα on epithelial cells and inhibiting nitric oxide production and iNOS mRNA expression [338]. However, the utility of antibiotics as primary, or adjuvant, therapeutic agents for CD treatment is controversial [339]. Uncontrolled trials with objective outcome measures, seen in the context of the randomized controlled trials, suggest that antibiotics, as single agents or in various combinations and with different course lengths, may be of clinical benefit and probably exert an anti-inflammatory effect in active CD [338]. The best-established indication for antibiotic treatment in IBD is pouchitis, often in combination with probiotic cocktails to maintain remission [338,340]. Single antibiotics have some benefit for CD colitis treatment as well as septic complications, such as abscesses and fistulae, and in preventing postoperative recurrence [341,342]. In addition, the role of oral vancomycin and gentamicin in very early-onset IBD has given interesting preliminary results [343]. Broad antibiotic combinations may ameliorate the outcomes, although long-term efficacy may be reduced by development of adverse events caused by long-lasting changes in microbiota composition and the emergence of antibiotic resistance [60]. Administration of nonabsorbable antibiotics, such as rifaximin, acting strictly on the gut lumen, may avoid systemic side effects [344]. The use of antibiotics in ulcerative colitis (UC) is more controversial, although promising results have been obtained with broad-spectrum oral antibiotic cocktails for the treatment of acute severe colitis and chronic persistent UC [338].

### 5.2. Probiotics and Prebiotics

Probiotics, microorganisms similar to the beneficial bacteria present in the human gut, have been widely studied in many gastrointestinal diseases. The most studied probiotics for human use belong to the *Lactobacillus*, *Bifidobacterium*, and Saccharomyces species and are available in a variety of over-the-counter (OTC) or prescription forms (capsules, packets, or food supplements) [345,346]. In the gastrointestinal tract, probiotics may contribute to the maintenance of the immunologic equilibrium and represent a promising approach in a variety of gastrointestinal, pancreatic, and liver disorders, but, to date, solid clinical data are confined to the treatment of infections, IBD, and IBS [346]. The rationale for the use of probiotics in IBD is based on accumulating evidence suggesting that the endogenous intestinal microbiota plays a crucial role in its pathogenesis. However, thus far, data from clinical trials suggest that the efficacy of probiotics in IBD treatment is rather low and limited to UC patients, since CD patients do not seem to have any benefit from probiotic administration [36,347]. Evidence from a number of controlled trials featuring different probiotic organisms, including nonpathogenic *E. Coli*, *S. boulardii*, *Lactobacillus,* and *Bifidobacterium*, is suggestive for the efficacy of probiotics in maintaining remission in UC and in treating mild to moderate flare-ups, while other studies have been less favorable [348,349]. Such discrepancies may be explained by the different study design and outcome, strain and doses of probiotics, and patient phenotype [342]. Probiotic formulations containing multiple species with different combinations of microorganisms, warranting microbial diversity, are more commonly applied. The VSL#3, a widely studied and commercialized combined preparation that contains eight strains of lactic acid-producing bacteria (*L. plantarum, L. delbrueckii subsp. bulgaricus, L. casei, L. acidophilus, B. breve, B. longum, B. infantis,* and *S. salivarius subsp. Thermophilus)* is efficacious in reducing active inflammation and in maintaining remission in patients with mild-to-moderate UC [348,349]. Recently, a meta-analysis study concluded that VSL#3 is effective in preventing pouchitis episodes [349]. VSL#3 has been shown to increase regulatory cytokines levels and to reduce pro-inflammatory cytokines, TLRs, NF-κB, and TNF-α expression [350]. Although the mechanism of action of probiotics is uncertain, it is likely that several mechanisms operate together. Probiotics prevent opportunistic pathogens gut colonization by blocking their attachment to the epithelium and by producing molecules which inhibit pathogen replication, such as lactic, propionic, and acetic acid, and bacteriocins. In addition, probiotics have different modulatory effects on the host innate and adaptive immune system function, including macrophage, NK and cytotoxic T cell activation, regulation of IgA production and cytokine-expression profile, and stimulation of TLRs [351]. The ability of probiotics to activate specific opioid and cannabinoid receptors in the gut makes them useful to reduce visceral pain in patients with IBS and chronic IBD [352]. By restoring the normal gut microbiota, probiotics may extend their influence from the gut to the brain along microbiota–gut–brain axis, fostering beneficial effects in the treatment of brain disorders, including autism spectrum disorder, Parkinson’s disease, multiple sclerosis, and mood disorders [353]. In this context, probiotics might be effective, not only in the treatment of persistent gastrointestinal symptoms but also of IBD-associated anxiety and depression [21]. However, up to now, there is insufficient support from clinical studies to suggest that probiotics may provide benefits for the relief of depressive symptoms, in non-IBD patients [354].

Administration of prebiotic dietary substrates, such as oligosaccharides and fibers, targeting microbiome composition, is a further microbiome-based approach, which, however, has given unsatisfactory results in IBD treatment thus far [339]. Fermentable oligosaccharides, disaccharides, monosaccharides, and polyols (FODMAPs) are resistant to digestive enzymes produced by the human body, while remaining susceptible to colonic anaerobic bacteria, which metabolize the oligosaccharides to SCFAs and selectively stimulate the growth of Bifidobacteria [355]. FODMAPs ingestion results in increased luminal water and gas volume, leading to the perception of pain, but only in selected patients with visceral hypersensitivity, and can induce gut symptoms in quiescent IBD [356,357]. In this perspective, in IBD patients, a low FODMAP diet may be potentially efficacious, as demonstrated in a randomized-controlled study by the improvement of gut symptoms in 81% of patients with IBD compared to controls [358,359]. A recent randomized, small placebo-controlled dietary advice trial of low FODMAP diet in quiescent IBD reports the improvement in some gastrointestinal symptoms and in the patients’ health-related quality of life [360]. Notwithstanding a reduction in Bifidobacteria and *F prausnitzii* abundance, this 4-week low FODMAP diet did not adversely affect disease activity.

A new frontier in the field of gut microbiota-based therapy for IBD is represented by the production of next-generation probiotics, which represent a new class of live biotherapeutic products addressing specific mechanisms of disease. Many bacterial species have been evolutionarily selected for metabolic function within the mammalian gastrointestinal tract, and the probiotic organisms exploited, thus far, have a long history of safe use in humans [213]. Engineered live biotherapeutic products may be designed to sense and respond to stimuli within the gut environment and represent an opportunity to influence host biology in situ. *F. prausnitzii* strains, owing to their anti-inflammatory and protective effect in the gut, are gaining interest as potential next-generation probiotics [52,361]. These beneficial microbes and their metabolites are being considered as therapeutic agents in treatment of IBD [362]. A further advantage of live biotherapeutic products is the possibility to obtain high concentrations of metabolites, such as SCFAs, TRP metabolites, and bile acids, so called “postbiotics”. Metabolite-based interventions are therapeutically attractive since these small molecules may reach physiologically high concentrations, retain a low potential for toxicity, and their administration follows the principles of pharmacokinetics, being suitable for different routes of administration and for attaining a desired systemic concentration [363].

### 5.3. Fecal Microbiota Transplantation

After its successful use in treating recurrent *C. difficile* infection, fecal microbiota transplantation (FMT) demonstrated significant efficacy in restoring intestinal microbial balance, leading to expand its use to treat local and systemic illnesses associated with gut dysbiosis [364]. To date, FMT is an established treatment for *C. difficile* infection and is also being considered for other gastrointestinal diseases such as IBD, IBS, hepatic steatosis, and hepatic encephalopathy [357]. Evidence for the efficacy of FMT in the treatment of IBD is controversial with some promising data for the induction of remission in UC and CD, as demonstrated by a systematic review of the literature and meta-analysis [365]. In a multicenter, double-blind, randomized, placebo-controlled trial, an intensive-dosing, multidonor FMT induced clinical remission and endoscopic improvement in UC and was associated with increased microbial diversity, which related with the outcome in FMT-treated patients with respect to patients assigned to placebo [366]. Accordingly, 1-week treatment with anaerobically low-intensity donor FMT resulted in a higher likelihood of remission at 8 weeks in adult patients with mild-to-moderate UC, compared to autologous FMT [367]. In mild-to moderate UC pediatric patients responding to FMT, the microbial, virome and metabolomic profiles changed post-FMT, moving towards the donor profile [368]. Notably, post-FMT, fecal concentrations of some microbial metabolites changed, with increased levels of butyrate, which were correlated with clinical improvement. The importance of donor screening and selection is fundamental for the success and safety of FMT therapy. In UC patients, FMT elicited fever and increased levels of C reactive [369]; furthermore, disease flares have been reported in patients with UC or CD after FMT [370,371]. Potential adverse events comprise short-term and long-term events, with short-term events being related either to the method of FMT delivery or the FMT itself or both. Although the long-term consequences of FMT treatment remain unclear, the availability of highly screened and catalogued stool banks, as well as of the advent of FMT capsule, allows for a safer and more simplified treatment [371]. However, several issues, including the different methods of delivery, dose, and frequency of administration and the influence of donor factors still hamper the evaluation of FMT efficacy, and standardized protocols are expected to emerge [372].

## 6. Future Perspectives and Conclusions

It is now well ascertained that dysbiosis highly impacts on immune, neuronal, and endocrine responses in the host, representing a fundamental contributor to IBD pathogenesis. Alterations of the microbial composition and load may concur to the development of local response to inflammation but may also extend their influence to more distant sites through the gut–brain axis, giving rise to psychiatric manifestations, frequently reported in IBD patients and negatively impacting on the patient’s mental status. Although a clear-cut demonstration of a causative relationship between dysbiosis and IBD has not been provided yet, in recent years, efforts in the field of metabolomics techniques in conjunction with observational and experimental data have highlighted the mechanistic contribution of microbial metabolites, also defined as “postbiotics”, to the pathogenesis of IBD. In the present dissertation, we have shown that SCFAs, secondary bile acids, and TRP metabolites may have neural, immune, and endocrine control of inflammation, but also of the mental health status during chronic intestinal inflammation.

Indeed, numerous reports highlight that microbial metabolites may influence not only the immune response, that ultimately drives the phenotypic characteristics of IBD but also gut–brain responses, influencing behavior and emotional functions. In this context, microbial metabolites may control bidirectional communication pathways that connect increased perceived stress with increased peripheral injury in IBD, establishing a bidirectional relationship between stress and IBD symptoms. However, up to now, most of the available evidence derives from preclinical studies, and only few clinical trials have evaluated the beneficial effect of microbiota-based therapies on the mental status of IBD patients.

A fundamental issue that needs to be solved concerns the possibility that changes in gut microbial ecology and function and the involvement of specific bacterial species can be predictive for relevant clinical questions, including stress-related behavior, inherent to IBD. In this context, the available clinical studies are retrospective studies, examining the microbiota composition after the onset of the disease, and thus, a more comprehensive understanding of the pathophysiological relevance of the saprophytic microflora and its metabolites in IBD involves the conduction of large-scale, highly controlled prospective clinical studies. Since IBD patients are represented by genetically and clinically defined subpopulations with highly specific microbial composition and function, the possibility to evaluate the patient microbial metabolomic profile may, indeed, represent a predictive clinical tool fostering the basis for a personalized microbiome-based therapy. A new frontier in the field of microbiome-based therapy for IBD is represented by direct administration of postbiotics with the dual advantage of allowing control of host homeostasis and correcting the negative effects of dysbiosis. In this regard, a promising strategy will be to combine different metabolomic, metagenomic, metatranscriptomic, and proteomic methodological approaches to enable a more comprehensive characterization of the complex microbial ecosystem and the relevant metabolites to verify their potential efficacy as adjuvant in the therapy of IBD and the related gut–brain axis disorders.

## Figures and Tables

**Table 1:** Main bacterial changes in the Phylum, Genus, and Species levels of the human microbiome associated with inflammatory bowel diseases (IBD).

Level	Bacteria	Variation	References
**Phylum**	*Firmicutes *	decrease	[41,65,66,67,68,69,70,71]
	*Bacteroidetes*	decrease	[41,65,66,67,68,69,70,71]
	*Proteobacteria* *(Enterobacteriaceae)*	increase	[41,65,66,67,68,69,70,71,72]
	*Actinobacteria*	increase	[41,65,68]
**Genus**	*Bacteroides *	decrease	[73,74,75,76]
	*Lactobacillus*	decrease	[73,74,75,76,77]
	Eubacterium	decrease	[73,74]
	*Clostridium* spp.	decrease	[55,56,78,79]
	*Fusobacterium* spp.	increase	[80,81,82]
**Species**	*Faecalibacterium prausnitzii*	decrease	[51,52,54,79,83]
	*Roseburia hominis*	decrease	[83,84]
	*Bifidobacterium adolescentis*	decrease	[79]
	*Dialister invisus*	decrease	[79]
	*Escherichia coli AIEC*	increase	[48,51,85,86]
	*Ruminococcus gnavus*	increase	[58,79]
	*Mycobacterium avium subsp. Para-tuberculosis*	increase	[87,88]

**Table 2:** Examples of cellular/tissue targets for microbial metabolites along the microbiota gut–brain axis involved in the intestinal inflammatory response.

Metabolite/ Teatment	Cell Type/Tissue	Findings	Reference
**SCFAs**			
Administration of a SCFA smixture (os)	Mouse colon mucosal Tregs	Regulation of cell function and size with protective effect against colitis via FFAR-2	[227]
Administration of n-butyrate (os)	Mouse colon mucosal macrophages	Downregulation of LPS-induced proinflammatory mediators, including nitric oxide, IL-6, and IL-12, via HDAC inhibition	[228]
High-fiber feeding	Mouse colon epithelial cells	Promotion of NLRP3 inflammasome-mediated IL-18 release contributing to gut homeostasis and protection from colitis via FFAR-2 and GPR109A	[230]
Administration of n-butyrate (ip)	Mouse hippocampus, prefrontal cortex	Anti-depressant effect associated with short-term HDAC inhibition and increased BDNF levels (only in prefrontal cortex)	[238]
Endogenous SCFA levels	Mouse colon mucosa, stools	Reduction of SCFAs and *Lactobacillus spp* fecal content, increase in GPR109A colonic expression after stressor exposure during infection with *Citrobacter Rodentium* underlying gut inflammation	[241]
Administration of a SCFAs mixture (os)	Mouse hypothalamus, hippocampus, colon mucosa	-Decreased stress-induced CRF expression in the hypothalamus, and CRF-receptor 1 in the hippocampus -Amelioration of psychosocial stress-induced responses -Anti-depressant and anxiolytic effects -Rescue of stress-induced enhancement of colonic mucosa permeability	[242]
**Bile acids**			
Administration of the FXR agonist, INT-747, (i.p.)	Mouse colon mucosal macrophages	-Reduced activation and release of IL-1β, IL-2, IL-6, IFN-γ, and TNF-α -Amelioration of TNBS- and DSS-colitis	[253]
-Administration of INT-747 (os, in mice) -INT effect in vitro on LMPCs cultures	-Mouse colon epithelial cells - IBD patient’s LMPCs	-Amelioration of TNBS- and DSS-colitis, rescue of the mouse epithelial barrier function and reduced proinflammatory cytokine release in the mouse colon -Decrease TNF-α secretion in LMPCs from patients with IBD,	[254]
Administration of ciprofloxacin (i.p.) or oleanolic acid (os) to activate TGR5	Mouse and human colon mucosa	-Increased levels of TGR5 in the mouse colon after TNBS- and DSS-colitis, and in the colon of IBD patients -reduced colonic TLR4-mediated TNF-α secretion in TNBS-treated mice	[258]
-Administration of TGR5 agonists -BAR501, (os) (ref 352) -BTA (os) (ref 253) -Several TGR5 agonist in vitro (ref 254)	-Mouse colon mucosa macrophages -LMPCs from IBD patient’s intestinal mucosa	-Amelioration of the severity of colitis in TNBS, DSS and oxazolone-treated mice -Shift of intestinal macrophages from a classically activated proinflammatory M1 to an anti-inflammatory M2 phenotype in mice -reduced colonic expression of pro-inflammatory genes (TNF-α, IFN-γ, IL-1β, IL-6, and CCL2 mRNAs) and increased expression of IL-10 and TGF-β mRNAs in mice -reduced TNF-α release from proinflammatory macrophages isolated from IBD patient’s LMPCs	[260,261,262]
Administration of UDCA, TUDCA, GUDCA (os)	Mouse colon	-Amelioration of the severity of colitis in DSS-treated mice -Normalization of *Firmicutes* to *Bacteroidetes* ratio after colitis-induced dysbiosis -Prevention of *Clostridium cluster* XIVa loss and increased abundance of the protective species, *Akkermansia muciniphila*	[263]
**Tryptophan metabolites**			
**Kynurenines**			
IDO1	-Mouse colon crypt epithelial cells -Mouse colon lamina propria infiltrating APCs	- IDO1 levels of expression are fundamental to reduce Treg immune suppression and the severity of the inflammatory injury after TNBS-, DSS- and T-cell transfer-colitis	reviewed in ref [291]
Endogenous IDO1 and KynA	Epithelial cells and LMPCs from IBD patients’ biopsies	Increased IDO1 expression in epithelial and mononuclear cells positively related to the severity of inflamed region	[293]
	UC patients’s serum and mucosa	Increased IDO mucosal expression and increased KynA serum levels, positively correlated with endoscopic inflammation	[294]
Exposure in vitro to KynA and synthetic analogues	Human monocytic isolated cells	Reduced production of proinflammatory TNF-α induced by *Staphylococcus aureus* and *Chlamydia pneumoniae*, via TSG-6 expression	[297,298]
Endogenous IDO1, TRP, KynA	Mouse brain, spleen, Peyer’s patches	-Psychological stress-induced IDO1 activity, blocked by anti-TNFα and IFNγ treatment, and increased plasmatic KynA/TRP ratio	[299]
Administration of KynA (i.v.)	Dog colon ENS	-KynA antagonized hypermotility and inflammatory changes (increased XOR and MPO activity) associated with transitory colonic obstruction, via enteric NMDA receptors	[300]
Administration of KynA and its analogue, SRZ-72 (i.v.)	Rat colon ENS	-Reduced proinflammatory cytokine release, nitrite/nitrate and nitrotyrosine formation after TNBS-induced colitis -Normalization of microcirculation and of the rate of bowel movements after TNBS-induced colitis NMDA receptors located on enteric neurons	[302]
Endogenous Kyn/TRP	Rat intestine and CNS	FMT from depressed patients to bacterial microbiota-depleted animals induced: -Increased anxiety-like and anhedonic behavior -Increased Kyn/TRP plasma ratio -Increased bowel movements -Reduced microbiota richness	[303]
Kyn/TRP and Kyn	Mouse colon and hippocampus	Kyn and Kyn/TRP and cytokine plasma levels increased after *Trichuris muris*-induced mild to moderate colonic inflammation associated with -anxiety-like behaviors and reduced hippocampal BDNF mRNA levels	[210]
**AhR ligands**			
-Endogenous AhR -administration of AhR agonist FICZ in vitro and ip	-UC and CD biopsies -Mouse colon	-Reduced AhR levels in DSS-treated mice colon and IBD biopsies -Reduced iIFNγ levels and IL-22 upregulation in patients’ LPMCs - Reduction of the severity of TNBS-, DSS and T-cell transfer-induced colitis in mice by Ficz -Downregulation of proinflammatory cytokines and upregulation of IL-22 in TNBS-treated mice which was reversed by FICZ antagonism -AhR activation by FICZ ameliorates DSS-induced colitis in mice via the MK2/p-MK2/TTP pathway	[329,336]
Administration of AhR agonist, ITE (ip)	Mouse colon	-Reduction of colitis severity in a humanized murine model of human CD4+ T cells transfer after TNBS administration -Increased CD39, Granzyme B, and IL-10-secreting human Treg cells isolated from colonic LPMCs.	[330]
Endogenous IAA	-Mouse colon and stools -IBD patients’ stools	-Susceptibility to colitis and altered microbiota in Card9 −/− mice associated with significant reduction of IAA fecal levels Reduced IAA and TRP levels and increased Kyn levels in fecal samples from IBD patients, particularly in those with CARD9 risk alleles associated with IBD	[331]
Endogenous and exogenous IPA	-IBD patient’s serum -Mouse colon and serum	-Reduced serum indole and IPA levels -Reduced serum indole and IPA levels after DSS colitis in mice -Improvement of DSS-colitis by exogenously administered IPA via epithelial IL-10 signaling	[332]
Endogenous IA	-Mouse colon goblet cells -Stools from CD and UC patients	-Administration of IA producing *Peptostreptococcus russellii* carrying the phenyllactate gene cluster (fldAIBC) to mice, reduced susceptibility to DSS, improved mucin producing goblet cell differentiation, promoted immune tolerance -reduced abundance of phenyllactate gene cluster in UC by metagenomic analysis	[335]

Abbreviations: Antigen-Presenting Cells, (APCs); betulinic acid (BTA); glycoursodeoxycholic acid (GUDCA), Lamina propria mononuclear cells LPMCs, tauroursodeoxycholic acid (TUDCA), ursodeoxycholic acid (UDCA).
